# Supplementary material for: Risk of Psoriasis in Patients with Polycystic Ovary Syndrome: A National Population-Based Cohort Study
Source: J Clin Med. 2020 Jun 22;9(6):1947. doi: 10.3390/jcm9061947 (PMC7357083; doi:10.3390/jcm9061947)
Supplement: Supplementary file 1 [file jcm-09-01947-s001.pdf]

STROBE Statement—Checklist of items that should be included in reports of ***cohort studies***

	**Item No**	**Recommendation**
**Title and abstract**	1	(*a*) Indicate the study's design with a commonly used term in the title or the abstract<br>*Yes, A National Population-Based Cohort Study*
		(*b*) Provide in the abstract an informative and balanced summary of what was done and what was found<br>*Yes, the incidence of psoriasis is higher in PCOS patients compared to that in control group.*
**Introduction**		
Background/rationale	2	Explain the scientific background and rationale for the investigation being reported<br>*Yes, the psoriatic patients have a higher risk of PCOS. Psoriatic patients with PCOS feature more severe skin lesion compared to those with psoriasis alone. But the risk of psoriasis in PCOS patients is largely unknown.*
Objectives	3	State specific objectives, including any prespecified hypotheses<br>*Yes, we proposed that the risk of psoriasis is higher in PCOS patients.*
**Methods**		
Study design	4	Present key elements of study design early in the paper<br>*Yes, this retrospective cohort study compared the incidence of psoriasis in PCOS patients with those patients free of PCOS.*
Setting	5	Describe the setting, locations, and relevant dates, including periods of recruitment, exposure, follow-up, and data collection<br>*Yes. In this study, we used the Longitudinal Health Insurance Database (LHID), which is a subset of the medical records in the NHI program including almost the entire Taiwan population. The dataset included all the medical records of randomly selected 1,000,000 Taiwanese from 2000 to 2012.*
Participants	6	(*a*) Give the eligibility criteria, and the sources and methods of selection of participants. Describe methods of follow-up<br>*Yes. Patients diagnosed as having PCOS (ICD-9-CM code 256.4) between 2000 and 2012 were recruited as the case group in this cohort study. The disease was identified by the presence of at least three related outpatient diagnoses or at least one related inpatient diagnosis to ensure the validity of disease diagnosis.*
		(*b*) For matched studies, give matching criteria and number of exposed and unexposed<br>*Yes. The control group included women free of PCOS and psoriasis matched to the participants of the PCOS group by age and index year through propensity score matching at the ratio of 1:4.*
Variables	7	Clearly define all outcomes, exposures, predictors, potential confounders, and effect modifiers. Give diagnostic criteria, if applicable<br>*Yes. The outcome is the occurrence of psoriasis. The exposure is PCOS. The potential confounders include insulin resistance related comorbidities in PCOS patients. In addition, age at the index year and index year were used for propensity score matching.*
Data sources/ measurement	8*	For each variable of interest, give sources of data and details of methods of assessment (measurement). Describe comparability of assessment methods if there is more than one group<br>*Yes, the data source is the Longitudinal Health Insurance Database (LHID). The assessment methods mainly relied on the diagnosis code for PCOS, psoriasis, and comorbidities. The assessment method is the same for the PCOS and control group.*
Bias	9	Describe any efforts to address potential sources of bias

		Yes. To avoid misclassification bias, the disease was identified by the presence of at least three related outpatient diagnoses or at least one related inpatient diagnosis to ensure the validity of disease diagnosis.
Study size	10	Explain how the study size was arrived at<br>*Yes. The database includes medical records of one million Taiwan people. This is a study based on national population.*
Quantitative variables	11	Explain how quantitative variables were handled in the analyses. If applicable, describe which groupings were chosen and why<br>*Yes. The patients were divided into three subgroups (<20 years, 20-50 years, >50 years) according to the age at the year of diagnosis. The age groups were divided by age of reproductive age and age of menopause. Because the incidence of psoriasis are higher at puberty and menopause.*
Statistical methods	12	(*a*) Describe all statistical methods, including those used to control for confounding<br>*Yes. The chi-square test was conducted to examine the differences in categorical variables between the control and PCOS groups. The mean ages were compared using Student's t-test. We obtained the cumulative incidence curve using the Kaplan–Meier method and assessed the difference between the groups using the log-rank test. Hazard ratios (HRs) were obtained using the Cox proportional hazards regression model.*
		(*b*) Describe any methods used to examine subgroups and interactions<br>*Yes. We also performed stratification analysis to elucidate the interaction between PCOS and comorbidities to investigate if it influences the incidence of psoriasis in the case and control groups.*
		(*c*) Explain how missing data were addressed
		(*d*) If applicable, explain how loss to follow-up was addressed
		(*e*) Describe any sensitivity analyses<br>*The statistical methods for PCOS and other diseases in NHID have been validated in previous reports [12-14].*
**Results**		
Participants	13*	(a) Report numbers of individuals at each stage of study—eg numbers potentially eligible, examined for eligibility, confirmed eligible, included in the study, completing follow-up, and analysed<br>*Yes. During the follow-up period, 4707 patients with PCOS and 18,828 controls were identified from the LHID. The mean follow-up times of the control and PCOS groups were 6.94 ± 3.53 and 6.99 ± 3.53 years, respectively.*
		(b) Give reasons for non-participation at each stage<br>*Yes. The NHI program almost covers the whole population in Taiwan. The participants leave the NHI program only after death or immigration to other countries and give up the nationality of Taiwan.*
		(c) Consider use of a flow diagram<br>*No.*
Descriptive data	14*	(a) Give characteristics of study participants (eg demographic, clinical, social) and information on exposures and potential confounders<br>*Yes. The demographic and clinical comorbidities for the case and control groups are listed in Table 1.*
		(b) Indicate number of participants with missing data for each variable of interest<br>*No.*
		(c) Summarise follow-up time (eg, average and total amount)<br>Yes. *The mean follow-up times of the control and PCOS groups were 6.94 ± 3.53 and*

		6.99 ± 3.53 years, respectively.
Outcome data	15*	Report numbers of outcome events or summary measures over time<br>*Yes. The incidence rates of psoriasis in the control and PCOS groups were 0.34 and 0.70 per 1000 person-years, respectively.*
Main results	16	(*a*) Give unadjusted estimates and, if applicable, confounder-adjusted estimates and their precision (eg, 95% confidence interval). Make clear which confounders were adjusted for and why they were included<br>*Yes. The PCOS group showed a higher risk of psoriasis by an HR of 2.07 (95% confidence interval [CI] = 1.25–3.43, P < .01) compared with the control group.*
		(*b*) Report category boundaries when continuous variables were categorized<br>*Yes. People aged more than 50 years were more likely to develop psoriasis by an HR of 14.13 (95% CI = 1.8–110.7, P < .05) relative to the population aged less than 20 years.*
		(*c*) If relevant, consider translating estimates of relative risk into absolute risk for a meaningful time period<br>*No.*
Other analyses	17	Report other analyses done—eg analyses of subgroups and interactions, and sensitivity analyses<br>*Yes. The cumulative incidence curve of psoriasis was higher in the PCOS group than in the control group (P = .004, log-rank test). In the analysis stratified by comorbidities, such as asthma, COPD, CLD, DM, hypertension, HLA, depression, and sleep apnea, the HR of psoriasis in the PCOS group did not change significantly.*
**Discussion**		
Key results	18	Summarise key results with reference to study objectives<br>*Yes. In the present analysis, three independent risk factors (advanced age, PCOS, and cancer) were identified by adjusted HRs using the Cox regression model, suggesting that they are substantial risk factors for psoriasis.*
Limitations	19	Discuss limitations of the study, taking into account sources of potential bias or imprecision. Discuss both direction and magnitude of any potential bias<br>*Yes. The use of the LHID however is associated with some limitations in the present study. First, a misclassification bias might result from the coding process by each physician and hospital in Taiwan for health insurance claims. Second, data on potential confounding factors, such as smoking, alcohol consumption, and socioeconomic status, are not available in the LHID and could not be analyzed in the present study. Nonetheless, COPD could be the proxy variable for smoking, which was adjusted and did not affect the HR of psoriasis in the present study. Third, detailed data about body mass index in the index year are not available. The confounding effect of obesity is not minimized in the present study.*
Interpretation	20	Give a cautious overall interpretation of results considering objectives, limitations, multiplicity of analyses, results from similar studies, and other relevant evidence<br>*Yes. The first three paragraphs in the section of Discussion tried to list the source of difference among previous studies using LHID for PCOS and insulin resistance related comorbidity.*
Generalisability	21	Discuss the generalisability (external validity) of the study results<br>*Yes, the LHID only recruited Taiwan people, and ethnicity is a confounding factor for the incidence of psoriasis. The results in the present study need be carefully interpreted to apply directly for the people living at other geographic regions.*
**Other information**		
Funding	22	Give the source of funding and the role of the funders for the present study and, if

	applicable, for the original study on which the present article is based<br>*Yes. This research was funded in part by Taiwan Ministry of Health and Welfare Clinical Trial Center, grant number MOHW109-TDU-B-212-114004, and Taiwan Ministry of Science and Technology, Clinical Trial Consortium for Stroke, grant number MOST 108-2321-B-039-003-. The funders had no role in the design of the study; in the collection, analyses, or interpretation of data; in the writing of the manuscript, or in the decision to publish the results.*

*Give information separately for exposed and unexposed groups.

Note: An Explanation and Elaboration article discusses each checklist item and gives methodological background and published examples of transparent reporting. The STROBE checklist is best used in conjunction with this article (freely available on the Web sites of PLoS Medicine at http://www.plosmedicine.org/, Annals of Internal Medicine at http://www.annals.org/, and Epidemiology at http://www.epidem.com/). Information on the STROBE Initiative is available at http://www.strobe-statement.org.
